# The plasma miRNome and venous thromboembolism in high‐grade glioma: miRNA Sequencing of a nested case–control cohort

**DOI:** 10.1111/jcmm.18149

**Published:** 2024-04-13

**Authors:** Friedrich Erhart, Georg Widhalm, Barbara Kiesel, Matthias Hackl, Andreas Diendorfer, Matthias Preusser, Karl Rössler, Johannes Thaler, Ingrid Pabinger, Cihan Ay, Julia Riedl

**Affiliations:** ^1^ Department of Neurosurgery Medical University of Vienna Vienna Austria; ^2^ TAmiRNA GmbH Vienna Austria; ^3^ Clinical Division of Oncology Department of Medicine I Medical University of Vienna Vienna Austria; ^4^ Clinical Division of Haematology and Haemostaseology Department of Medicine I Medical University of Vienna Vienna Austria

**Keywords:** cancer, high‐grade glioma, microRNA, venous thromboembolism

## Abstract

Patients with high‐grade gliomas are at high risk of venous thromboembolism (VTE). MicroRNAs (miRNAs) are small non‐coding RNAs with multiple roles in tumour biology, haemostasis and platelet function. Their association with VTE risk in high‐grade glioma has not been comprehensively mapped so far. We thus conducted a nested case–control study within 152 patients with WHO grade IV glioma that had been part of a prospective cohort study on VTE risk factors. At inclusion a single blood draw was taken, and patients were thereafter followed for a maximum of 2 years. During that time, 24 patients (16%) developed VTE. Of the other 128 patients, we randomly selected 24 age‐ and sex‐matched controls. After quality control, the final group size was 21 patients with VTE during follow‐up and 23 without VTE. Small RNA next‐generation sequencing of plasma was performed. We observed that hsa‐miR‐451a was globally the most abundant miRNA. Notably, 51% of all miRNAs showed a correlation with platelet count. The analysis of miRNAs differentially regulated in VTE patients—with and without platelet adjustment—identified potential VTE biomarker candidates such as has‐miR‐221‐3p. Therewith, we here provide one of the largest and deepest peripheral blood miRNA datasets of high‐grade glioma patients so far, in which we identified first VTE biomarker candidates that can serve as the starting point for future research.

## 1 BACKGROUND

Patients with cancer are at high risk of venous thromboembolism (VTE), which is a combined term for deep vein thrombosis (DVT) and pulmonary embolism (PE). The risk is especially high in patients with high‐grade gliomas; those patients have a VTE risk of up to 20% per year.^1^ Development of VTE in cancer patients is associated with additional morbidity and with increased overall mortality.^2^

The pathophysiology of VTE in cancer patients is multifactorial and associated with patient‐ and treatment‐related factors as well as with cancer biology.^2^ In the past years the molecular profile of malignant brain tumours has been shown to be a major determinator of the thrombotic risk in this type of malignancy. While patients with glioma that harbour a mutation in the isocitrate dehydrogenase (IDH)‐1 gene were found to be at very low risk of VTE,^3^, ^4^ intratumoral expression of the sialomucin‐like glycoprotein podoplanin was found as a major risk factor for VTE.^5^ Podoplanin is an agonist to the platelet activation receptor C‐type lectin receptor type 2 (CLEC‐2) and binding of podoplanin to CLEC‐2 induces platelet aggregation, suggesting a specific mechanism of thrombogenesis that is related to platelet activation. Interestingly, low platelet counts have also been found as a risk factor for VTE in glioma,^6^ and platelet counts of patients with podoplanin‐expressing tumours were found to be decreased, suggesting that platelets are consumed in patients with podoplanin‐expressing tumours due to continuous activation. Nevertheless, the influence of glioma biology on platelet turnover and activation is incompletely understood.

MicroRNAs (miRNAs) are small non‐coding RNAs that target and regulate messenger RNAs (mRNA) gene expression. As one miRNA can function as a master regulator of up to 200 mRNAs, miRNAs can substantially alter tissue states and phenotypes—including all aspects of coagulation processes.^7^, ^8^ In the peripheral blood plasma of high‐grade glioma patients, differences in miRNAs compared to healthy individuals can result from various sources. For instance, it is known that glioblastoma sheds exosomes containing miRNAs into the blood and that host cells can take up these exosomes.^9^ Further, driven by e.g. blood–brain‐barrier disruption, also cell‐free miRNAs derived from glioblastoma cells are reliably found in the peripheral blood,^10^ that are chemically highly stable.^11^ Importantly, also platelets, although anucleate, contain a plethora of miRNAs and these miRNAs are also found in the circulation, i.e. in plasma and serum samples.^12^ It was shown that specific platelet‐enriched circulatory miRNAs reflect platelet activation in‐vitro and in‐vivo,^13^ and plasma levels of certain miRNAs have been found in diseases that are associated with enhanced platelet activation, such as myocardial infarction or diabetes.^14^ In summary, given their presence in high‐grade glioma patient blood and their potency to also influence coagulation processes, it seems likely that miRNAs could be candidates for VTE biomarker development.

We investigated whether miRNAs are differentially regulated in patients with high‐grade glioma at risk of VTE by measuring plasma miRNAs in patients with WHO grade IV glioma (mainly glioblastoma) with and without VTE during their disease course in order to evaluate their role in the prothrombotic state in these patients and as potential biomarkers for risk prediction of VTE. For comprehensively mapping the full miRnome of all detectable plasma miRNAs, we relied on next‐generation deep RNA sequencing for that purpose.

## 2 METHODS

### 2.1 Study design and study population

A nested case–control study was performed within patients with WHO grade IV glioma that had been included in the Vienna Cancer and Thrombosis Study (CATS). The detailed study design of CATS can be found in previous publications^15^, ^16^ and is briefly outlined in the following.

### 2.2 The Vienna Cancer and Thrombosis Study

The Vienna Cancer and Thrombosis study (CATS) is a prospective, observational cohort study that started in 2003 at the Medical University of Vienna, Austria. CATS was designed with the primary aim to investigate risk factors for VTE in patients with various types of cancer. Patients with newly diagnosed or progressive cancer after remission were included and followed for a maximum period of 2 years. Primary endpoint of the study was occurrence of symptomatic VTE.

Written informed consent was obtained from each patient, a structured interview about the patients' medical history was performed and a single blood sample was drawn at the day of study inclusion. Follow‐up was performed regularly approximately every 3 months per postal questionnaire. The observation period started from the time of blood sampling and lasted for 2 years or until the occurrence of VTE, death, loss of follow‐up or withdrawal of informed consent.

No routine screening for VTE was performed. Diagnosis of VTE had to be confirmed by duplex sonography or venography for deep vein thrombosis (DVT), and by computed tomography or ventilation/perfusion lung scan for pulmonary embolism (PE), respectively. In patients who died during follow‐up, death certificates and, if available, autopsy findings had been checked for diagnosis of fatal PE. Ethical approval of the study was received from the institutional ethics committee (number EK126/2003 and EK419/2008) and the study has been conducted in accordance with the Declaration of Helsinki.

### 2.3 A nested case–control study within WHO grade IV glioma patients included in CATS

The patient cohort of the current study is based on data from a previous publication, which comprised all patients with primary malignant brain tumours that had been included into CATS from October 2003 until March 2014 of whom a formalin‐fixed and paraffin embedded (FFPE) brain tumour specimen was available for immunohistochemical staining. From this original cohort, which consisted of 213 patients, we selected all patients with WHO grade IV glioma (according to the diagnostic standard at the time of first diagnosis) for the current study, leading to a cohort of 152 patients. Of those, 24 (16%) developed VTE during the 2‐year observation period. From the remaining 128 patients who did not develop VTE during the follow‐up period we randomly selected 24 age‐ and sex‐matched controls. Four patients had to be excluded due to missing/insufficient plasma samples, leading to a final group size of 21 patients with VTE (=cases) and 23 without VTE (=controls).

### 2.4 Blood sampling

At the day of study inclusion, blood was collected by sterile venipuncture into plasma vacuum tubes (Vacuette®; Greiner Bio One, Kremsmünster, Austria) containing one‐tenth volume sodium citrate stock solution at 0.129 mM and processed within a time period of 2 h. Plasma was obtained by centrifugation at 3000 *g* for 10 min, and aliquotes were stored at −80°C until the time of analysis.

### 2.5 Total RNA isolation and small RNA‐Seq

Total RNA isolation was performed using 200 μL plasma together with the miRNeasy Mini Kit (Qiagen, Germany) according the instructions of the manufacturer. A mix of three synthetic spike‐in controls (miRCURY spike‐in Kit, Qiagen, Germany) was diluted 1:250 (to avoid excessive consumption of sequencing reads) and added to each sample for monitoring RNA extraction efficiency. Following chloroform extraction, precipitation and washing, total RNA was eluted from miRNeasy Mini columns using 30 μL nuclease‐free water. RNA was stored at −80°C until further processing.

Small RNA sequencing was performed in plasma samples by using the miND® Next‐Generation‐Sequencing (NGS) assay (TAmiRNA, Vienna, Austria), which was described in detail in a previous publication.^17^ The miND assay uses exogenous small RNA spike‐ins with design features that reduce sequencing bias and therefore allow unbiased quality control as well as normalization of small RNA‐seq data.^18^ One μl of miND spike‐in were mixed with 8.5 μL of total RNA and processed using the Realseq Biofluids small RNA library prep kit. Libraries were quality controlled using a DNA1000 chip (Agilent, United States), pooled at equimolar rate und purified using BluePippin 3% agarose cassettes (Sage Sciences, United States).

Next‐generation sequencing was performed on an Illumina NextSeq 550 in high output mode. Reads were demultiplexed according to the reverse primer index, adapter trimmed and filtered for reads shorter than 17 nucleotides.

### 2.6 Statistical analyses

Matching was performed by randomly drawing one control observation for each VTE patient from the whole set of controls with similar age and same sex.

NGS data processing and data analysis was performed using the miRNA NGS Discovery pipeline (miND) software,^19^ which annotates microRNAs and other non‐coding RNAs from small RNA‐seq data, and can perform group‐ or pair‐wise statistical analyses based on EdgeR. *p*‐Value from EdgeR based differential expression analysis were adjusted for multiple testing using Benjamini‐Hochberg false discovery rate.

RPM values of miRNAs were used for correlation against blood count parameters using spearman correlation and spearman rank sum test was used to estimate *p*‐values to which FDR adjustment was performed.

## 3 RESULTS

### 3.1 The study cohort comprised 21 patients who developed VTE during follow‐up and 23 matched patients without VTE

The final study cohort, that was assembled based on a nested age‐ and sex‐matching approach, consisted of 44 WHO grade IV glioma patients in whom peripheral blood samples were available for mapping the miRNome by means of small RNA sequencing. Of these, 21 patients had developed VTE during the follow‐up period (=cases) and 23 had not developed VTE during the follow‐up period (=controls). All patients were diagnosed with histologically confirmed WHO grade IV glioma. Detailed characteristics of the study population are listed in Table 1. The 21 thromboembolic events that occurred during the observation period consisted of 11 DVTs of the lower extremity, 9 PEs and one DVT of the upper extremity. PE was fatal in 1 case (4.7% of all the thromboembolic events).

**TABLE 1:** Baseline characteristics of study patients.

	Cases (VTE during follow‐up), *n* = 21	Controls (no VTE during follow‐up), *n* = 23
Median age at study entry, years (25th–75th percentile)	57 (48–67)	58 (48–67)
Female, *n* (%)	5 (23.8)	5 (21.7)
Median observation time, days (25th–75th percentile)	101 (23–180)	331 (172–474)
Platelet count, G/l, median (25t–75th percentile) *n*	217 (182–250)	226 (178–332)
Soluble *p* selectin, ng/mL, Median (25th–75th percentile)	51.5 (31.5–62.6)	36.7 (27.9–45.1)
Newly diagnosed glioblastoma, *n* (%)	19 (90.5)	21 (91.3)
Recurrent disease after remission, *n* (%)	2 (9.5)	2 (8.7)
Podoplanin expression level, *n* (%)		
No expression	1 (4.8)	5 (21.7)
Low	6 (28.6)	10 (43.5)
Medium	7 (33.3)	6 (26.1)
High	7 (33.3)	2 (8.7)
IDH1 mutation, *n* (%)	0 (0.0)	3 (13.0)

*Note*: Cases and controls were matched on age at study inclusion and biological sex.

### 3.2 Quality control parameters confirmed reliability of miRNA sequencing in all samples

**FIGURE 1:**
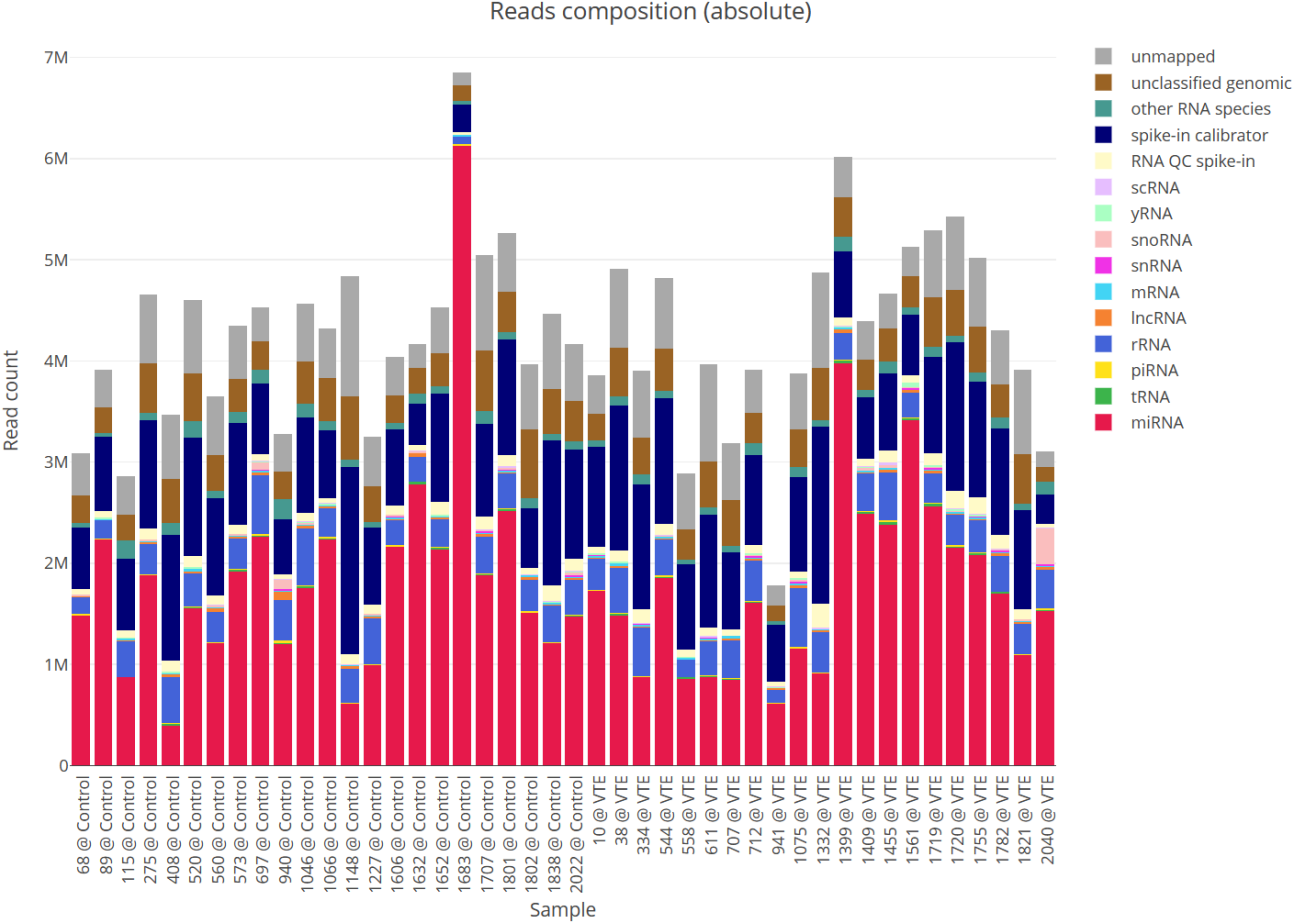
Classification of NGS reads in all analysed samples. Reads classification gives insights into the type and origin (i.e. composition) of all sequences obtained for each sample. After processing of the reads (adapter trimming, quality filtering, size filtering), all reads are mapped against various databases to categorize them. This is done in a hierarchical process, where reads are first mapped against the genome. Genome mapped reads are then mapped against known miRNA sequences and only those not identified as miRNAs get mapped against other databases for further classification. ‘Unclassified genomic’ indicates reads that were mapped against the genome but were not found in any of the RNA specific databases, while ‘unmapped’ are reads that could not be found in the given reference genome.

Small RNA sequencing was performed in all patients of the study cohort. As first analysis step, quality control measurements were performed using multiQC. We found that all fundamental parameters of quality were robust and solid. Sequencing depth ranged between 2 and 7 million reads. In all samples analysed, miRNA associated reads contributed on average 40% of the total reads. Figure 1 shows the overall distribution of all reads into various classes of small non‐coding RNAs for each sample. Further, as haemolysis might influence circulating miRNAs,^20^ values of lactate dehydrogenase (LDH) were analysed and no relevant indicators for haemolysis as a potential confounder were registered (see Figure S1).

### 3.3 Exploratory profiling identified the most abundant miRNAs

**FIGURE 2:**
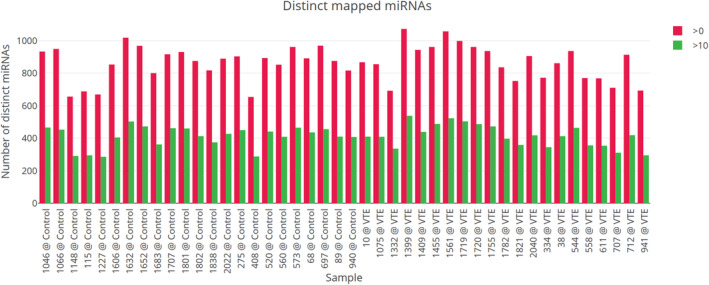
Number of distinct miRNAs. This graph shows the amount of distinct mature miRNAs identified in each sample.

Next, we aimed at mapping the overall miRNome found in the sequenced plasma. Within the individual samples, the number of distinct miRNAs identified which had a read count of at least 10, ranged from 286 to 538 (Figure 2). The mind® spike‐in calibrator fit had an R‐squared of at least 0.99 in all samples.^17^ Only miRNAs that showed more than 1 reads per 1 million microRNA reads (RPM) in at least 50% of samples were retained for subsequent analyses. This filtering step removes miRNAs that have a high CV but are only expressed in a too small number of samples to bear any statistical significance or biological relevance.

**FIGURE 3:**
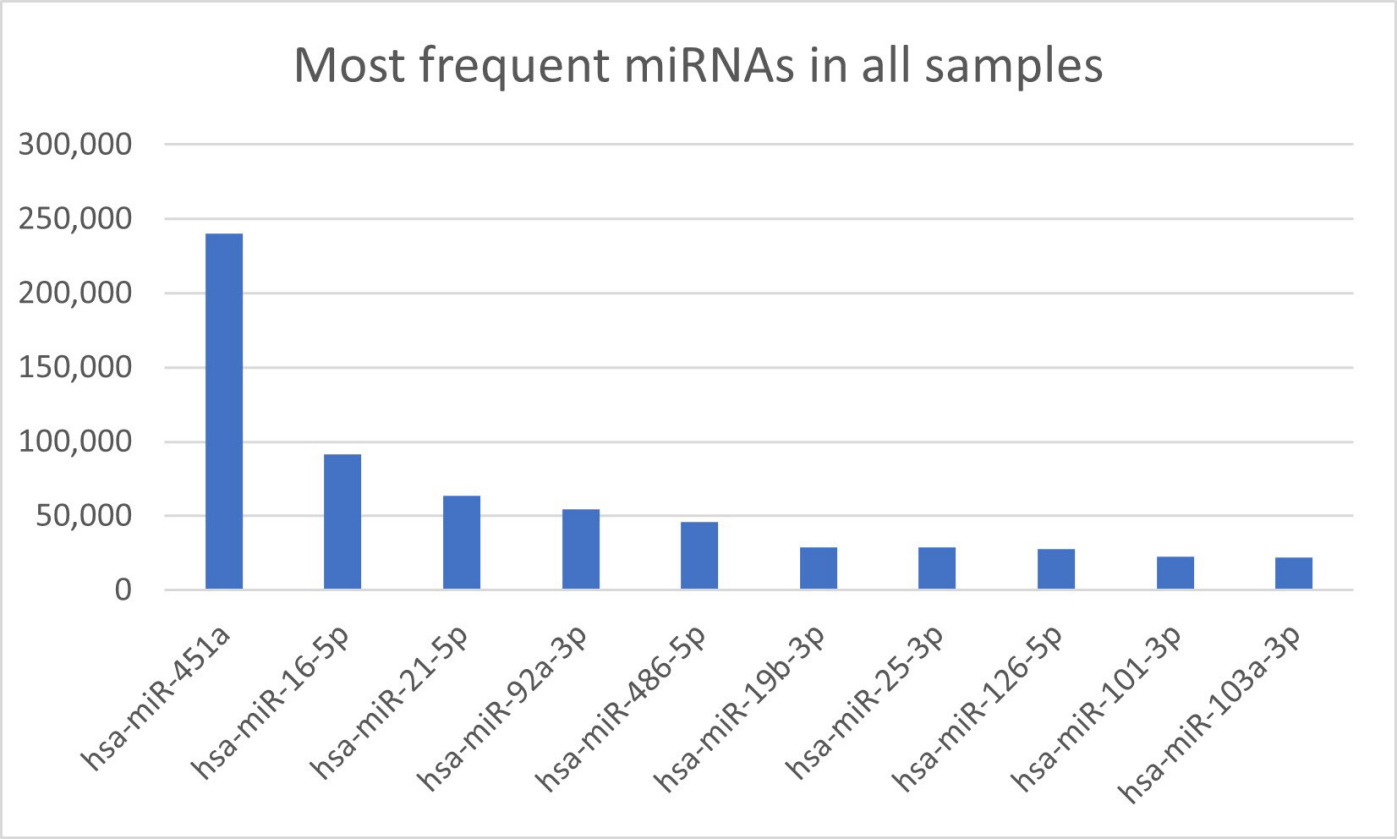
Ranking of the most abundant miRNAs in all samples. Here, the generally most frequent miRNAs are listed, with the average read count per 1 million mapped miRNAs on the y axis. miR‐451a sticks out as its frequency is more than double that of the next frequent miRNA, i.e. miR‐16‐5p.

Filtering of low abundant miRNAs resulted in a set of 328 miRNAs with good signal‐to‐noise ratio. The 10 miRNAs with the highest average RPM were hsa‐miR‐451a, hsa‐miR‐16‐5p, hsa‐miR‐21‐5p, hsa‐miR‐92a‐3p, hsa‐miR‐486‐5p, hsa‐miR‐19b‐3p, hsa‐miR‐25‐3p, hsa‐miR‐126‐5p, hsa‐miR‐101‐3p and hsa‐miR‐103a‐3p (Figure 3). They all had an RPM of more than 20,000. In our cohort, they represent the core miRNome found in the peripheral blood of patients with high‐grade gliomas. Of note, the miRNA at the very top in terms of average frequency, miR‐451a, stood out considerably as its abundance was more than twice as high as the next in line (miR‐16‐5p).

### 3.4 51% of miRNAs were significantly correlated with platelet count

In a first analysis of the connection of miRNAs with other factors, we assessed whether the measured miRNAs showed any association with variables of the complete blood count and with coagulation‐related parameters measured in peripheral blood. For these analyses, the total of 328 miRNAs that were robustly detectable in all samples, were used. The following peripheral blood parameters, which were available from previous studies, were analysed: haemoglobin (g/dL), platelet count (G/l), mean platelet volume (MPV, fl), leukocyte count (G/l), prothrombin time (PT, %), activated partial thromboplastin time (aPTT, seconds), fibrinogen (mg/dL), cogulation factor VIII activity (%), D dimer (μg/mL) and soluble *p* selectin (ng/mL). After correction for multiple testing, thirteen miRNAs were significantly correlated with haemoglobin, 10 miRNAs with leukocyte counts, five with PT, one with aPTT, eight with fibrinogen, one with factor VIII activity, 52 with D dimer and 12 with soluble *p* selectin. Twenty‐five miRNAs were significantly correlated with MPV. Most interestingly, however, 168 miRNAs were significantly (*p* < 0.05, FDR <0.1) correlated with the platelet count, i.e. 51% of the set of miRNAs with a robust read count (168/328). Thus, while most coagulation‐related peripheral blood variables showed a relation to only some miRNAs, platelet count stood out as it was related to approximately half of all identified miRNAs.

### 3.5 Unsupervised clustering of miRNAs showed no distinctive patterns

**FIGURE 4:**
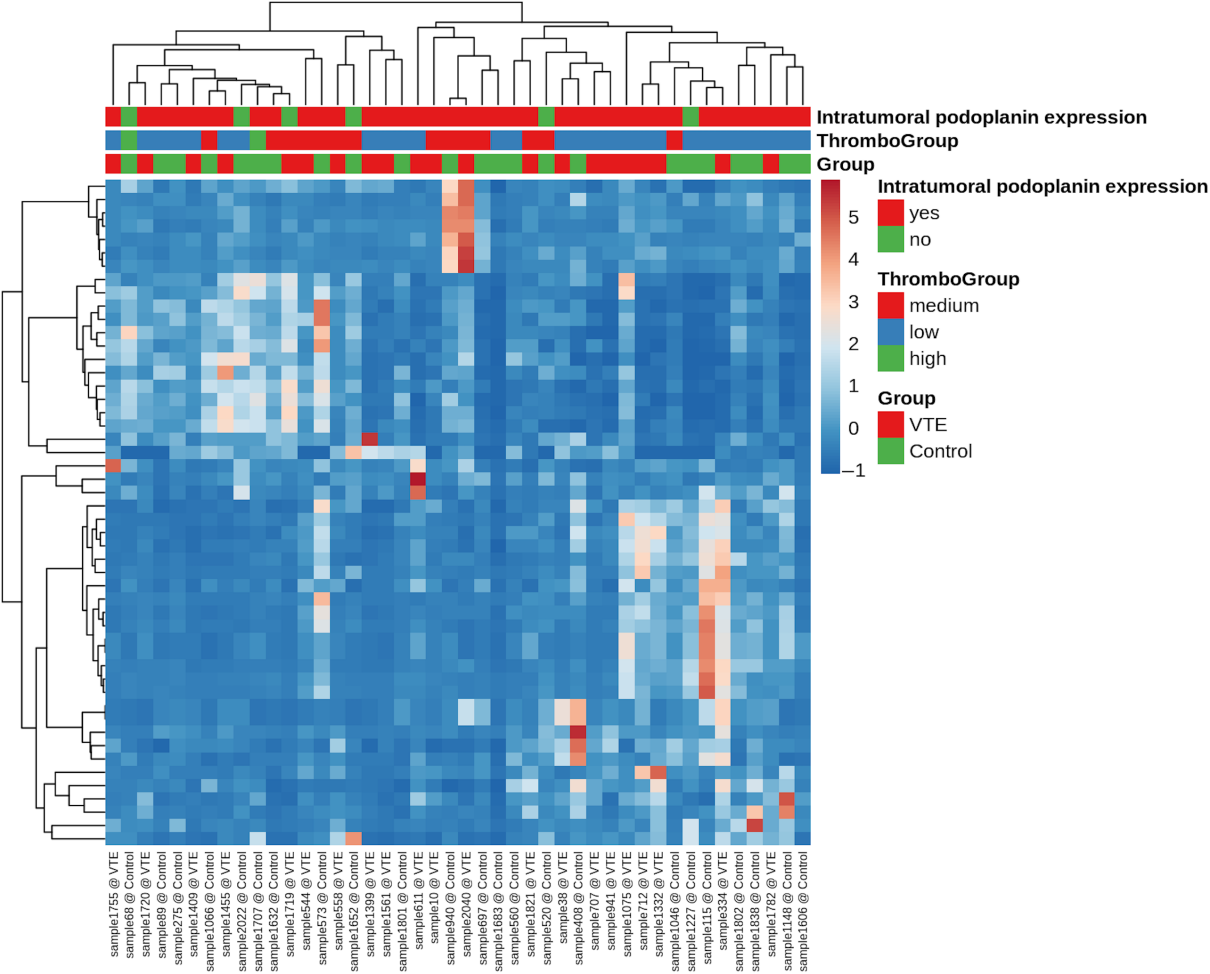
Heatmap—unsupervised clustering. This heatmap shows 439 miRNAs. Data are based on RPM normalized reads and scaled using the unit variance method for visualization in heatmaps. Clustering is done using the average method of heatmap calculating the distances as correlations. An additional filter was introduced to increase the robustness: only miRNAs that show an RPM in at least 1/*n* (groups) percent of samples (e.g. with 4 groups, the miRNA has to have an RPM value above 5 in at least 25% of the samples). This removes miRNAs that have a high CV but are only expressed in a too small amount of samples to bear any statistical significance or biological relevance.

Subsequently, we used unsupervised clustering to identify preeminent patterns in the miRNA data. These methods visualize and analyse data without knowledge of group assignments. As so‐called ‘unsupervised’ methods they allow to see if samples can already be grouped or clustered according to the given metadata—e.g. if all samples belonging to one group will also show similar expression profiles in a heatmap. Figure 4 shows the according heatmap for our miRNA data. We did not observe a clear pattern of miRNAs in groups of patients with similar platelet counts (grouped into terciles), podoplanin expression (any kind of Podoplanin expression vs. no Podoplanin expression) or VTE cases versus controls. Similarly, principal component analysis (PCA) and t‐stochastic neighbour embedding (t‐SNE) did not lead to definitive patterns with respect to the above‐mentioned parameters (Figure S2 and Figure S3).

### 3.6 Intratumoral podoplanin expression level was associated with a specific plasma miRNA profile

Next, we aimed at specifically mapping the association of intratumoral podoplanin with blood miRNAs. Podoplanin expression was determined by immunohistochemical staining of brain tumour tissue samples; these data were available from a previous study.^5^ MiRNA profiles in relation to intratumoral podoplanin expression (yes—tumour sample showed any kind of podoplanin expression vs. no—tumour sample stained negative for podoplanin) were explored. Table 2 shows differentially regulated miRNAs between patients with podoplanin positive versus podoplanin negative tumours (*p* < 0.05). As analyses were exploratory, no correction for multiple testing was performed. For the top up‐ or downregulated miRNAs, we then performed a target prediction analysis to explore likely target mRNAs. Table S1 gives the respective pathways identified.

**TABLE 2:** Differentially regulated plasma miRNAs (*p* < 0.05 without correction for multiple testing) between patients with podoplanin positive tumour samples (*n* = 6) versus patients with tumour samples, which did not express podoplanin (*n* = 38).

miRNA	logFC	*p*‐Value
Downregulated in patients with podoplanin positive vs. negative tumours		
hsa‐miR‐380‐3p	−1.0417	0.0436
hsa‐miR‐143‐3p	−0.9224	0.0162
hsa‐miR‐654‐3p	−0.8585	0.0434
hsa‐miR‐4454	−0.6497	0.0222
hsa‐miR‐1843	−0.5903	0.0434
hsa‐miR‐574‐3p	−0.5428	0.0396
hsa‐miR‐195‐5p	−0.4636	0.0474
Upregulated in patients with podoplanin positive vs. negative tumours		
hsa‐miR‐96‐5p	0.8713	0.0434
hsa‐miR‐32‐5p	0.5535	0.0434
hsa‐miR‐362‐3p	0.5401	0.0271

### 3.7 Exploratory screening identified miRNA biomarker candidates for risk prediction of VTE

**FIGURE 5:**
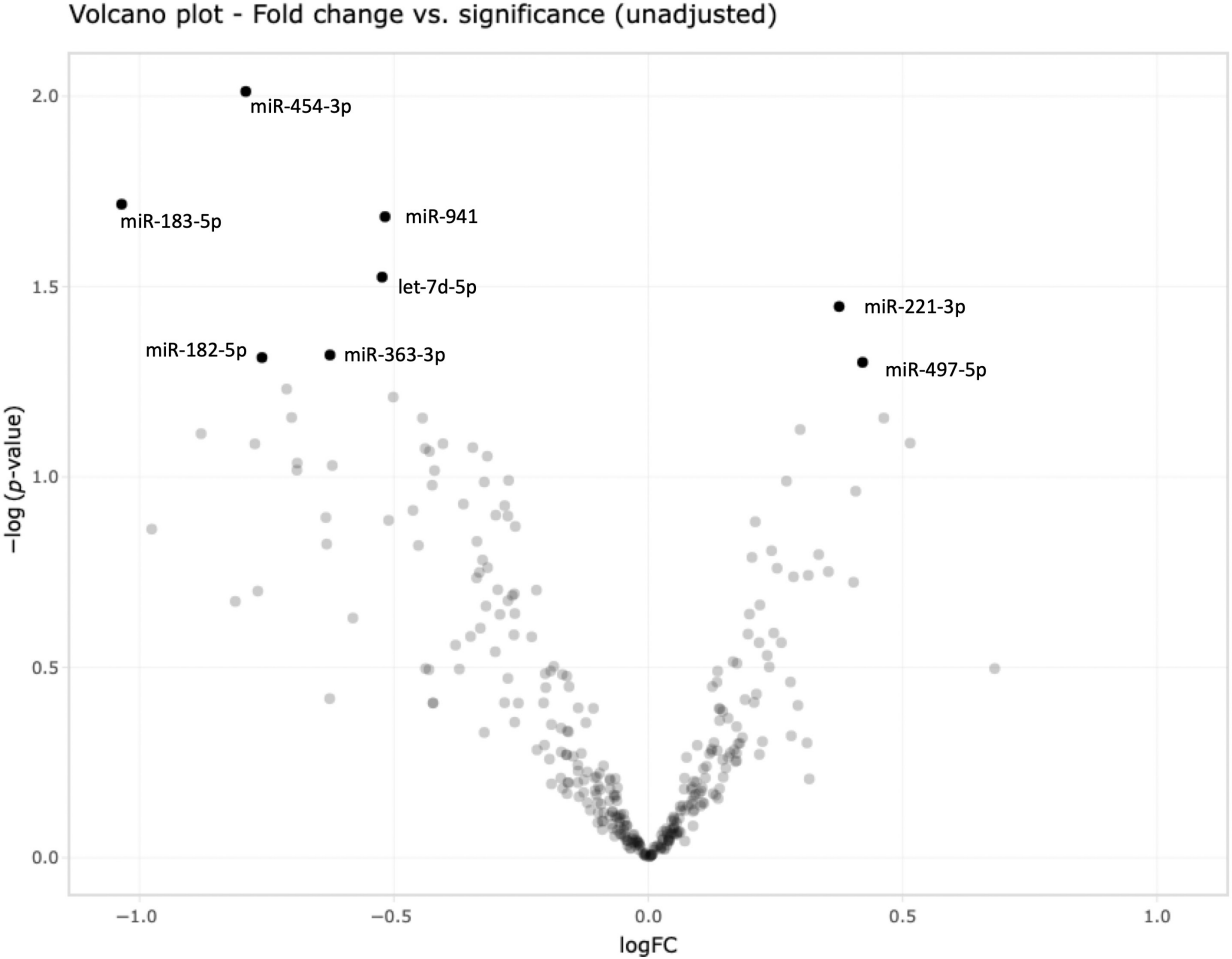
Volcano plot as exploratory analysis for differentially regulated miRNAs in VTE cases versus control (i.e. non‐VTE patients). This graph visualizes the relation of the logFC (how much did a miRNA change in the controls vs. cases) and the statistical significance of this change. miRNAs higher up have a smaller *p*‐value, while miRNAs more to the left or right of the centre, show a greater differential expression. Black dots indicate *p*‐value < 0.05 (unadjusted).

Finally, we investigated whether miRNA profiles differ between patients who developed VTE versus who did not develop VTE during the follow‐up period. For that, the registered miRNome of patients *without* VTE event was compared with patients *with* such an event (see Figure 5). In an exploratory screening before correction for multiple testing, we found several miRNAs significantly up‐ and downregulated in VTE cases versus controls, respectively (Table 3A). Among the downregulated miRNAs were miR‐182‐5p and miR‐363‐3p, among the upregulated was e.g. miR‐497‐5p. In a subsequent additional exploratory analysis, we adjusted for platelet counts, as we had observed a strong correlation between approximately half of miRNAs with platelet count. For that purpose and in light of the present sample size, we grouped the patients into three groups according to terciles of platelet count. In this additional analysis, we again found several miRNAs differentially expressed in controls versus cases (Table 3B), among them e.g. miR‐224‐5p and miR‐139‐3p. A total of five miRNAs were significantly differentially regulated in *both* analyses (before and after adjustment for terciles of platelet count): miRNAs miR‐183‐5p, miR‐454‐3p, has‐let‐7d and miR‐941 were significantly downregulated in VTE cases compared to controls, while miRNA 221‐3p was significantly upregulated, respectively. Again, as analyses were exploratory, no correction for multiple testing was performed.

**TABLE 3A:** Differentially regulated miRNAs (*p* < 0.05; without correction for multiple testing) between VTE cases and controls.

miRNA	logFC	*p*‐Value
Downregulated in VTE cases compared to controls		
hsa‐miR − 183‐5p	−1.035	0.01922
hsa‐miR‐454‐3p	−0.7913	0.009718
hsa‐miR‐182‐5p	−0.7594	0.04856
hsa‐miR‐363‐3p	−0.6258	0.04783
hsa‐let‐7d‐5p	−0.5233	0.02986
hsa‐miR‐941	−0.5175	0.02073
Upregulated in VTE cases compared to controls		
hsa‐miR‐497‐5p	0.4212	0.04999
hsa‐miR‐221‐3p	0.3751	0.03569

*Note*: Yellow highlights miRNAs differentially regulated (*p* < 0.05) (before and after adjustment for terciles of platelet count).

**TABLE 3B:** Differentially regulated miRNAs (*p* < 0.05; without correction for multiple testing) between VTE cases and controls, adjusted for terciles of platelet count.

miRNA	logFC	*p*‐Value
Downregulated in VTE cases compared to controls		
hsa‐miR‐183‐5p	−0.8733	0.04187
hsa‐miR‐454‐3p	−0.819	0.00864
hsa‐miR‐941	−0.5523	0.01697
hsa‐let‐7d‐5p	−0.5482	0.01965
hsa‐miR‐20b‐5p	−0.5221	0.04742
hsa‐miR‐503‐5p	−0.5016	0.04131
hsa‐miR‐18a‐3p	−0.4637	0.04498
hsa‐miR‐106a‐5p	−0.4553	0.04708
hsa‐miR‐151b	−0.3468	0.03685
hsa‐miR‐101‐3p	−0.3281	0.04246
Upregulated in VTE cases compared to controls		
hsa‐miR‐224‐5p	0.9032	0.001191
hsa‐miR‐139‐3p	0.6326	0.01868
hsa‐miR‐221‐3p	0.4414	0.01882
hsa‐miR‐598‐3p	0.4401	0.02148
hsa‐miR‐1271‐5p	0.4237	0.02154
hsa‐miR‐30d‐3p	0.3577	0.0421
hsa‐miR‐126‐5p	0.349	0.0461

*Note*: Yellow highlights miRNAs differentially regulated (*p* < 0.05) (before and after adjustment for terciles of platelet count). Pathway prediction analysis of VTE‐associated miRNAs identified mostly cancer‐related biological processes.

### 3.8 Pathway prediction analysis of VTE‐associated miRNAs identified mostly cancer‐related biological processes

Finally, to provide a first indication of genes and pathways regulated by the potentially VTE‐associated miRNAs, we concluded our work via performing a Diana mirPath pathway prediction analysis. We focused on miRNAs identified in both calculations, i.e. before and after platelet adjustment. For the top upregulated miRNA in VTE patients (hsa‐miR‐221‐3p), the algorithm predicted that the most affected pathways are e.g. p53 signalling or PI3K‐Akt signalling. For the top downregulated miRNAs in VTE patients (hsa‐miR‐183‐5p, hsa‐miR‐454‐3p, hsa‐miR‐941, hsa‐let‐7d‐5p), the algorithm identified e.g. the pathways proteoglycans in cancer and TGF‐beta signalling. Table 4 shows these predicted target pathways. The majority of them are cancer‐associated and not primarily coagulation‐associated.

**TABLE 4:** Pathway prediction analysis of the top differentially regulated genes in VTE cases versus controls.

KEGG pathway	*p*‐Value	Number of genes	Number of miRNAs
Targets of miRNAs downregulated in patients with VTE vs. controls (synthesis from before and after platelet adjustment)			
Adherens junction	3.22648498637e‐08	35	4
Proteoglycans in cancer	1.22267153464e‐07	66	4
TGF‐beta signalling pathway	5.81672422085e‐06	24	4
Hippo signalling pathway	5.81672422085e‐06	47	4
Viral carcinogenesis	3.14644659489e‐05	60	4
Targets of miRNAs upregulated in patients with VTE vs. controls (synthesis from before and after platelet adjustment)			
Fatty acid elongation	0.000375246094209	1	1
p53 signalling pathway	0.00446898150781	12	1
Hippo signalling pathway	0.00881624923835	12	1
Viral carcinogenesis	0.0119332032684	20	1
PI3K‐Akt signalling pathway	0.0246495631159	29	1

## 4 DISCUSSION

Prevention of VTE remains a major challenge in patients with cancer. The development of VTE results not only in additional morbidity for cancer patients but also frequently leads to interruption and delay of anti‐neoplastic treatment. Moreover, VTE treatment requires therapy with full dose‐anticoagulation, which is associated with an increased risk of major bleeding, and intracranial bleeding is the most feared complication.^21^ Therefore, research of the last decades focused on the identification of risk factors for cancer‐associated VTE with the aim to identify those patients at highest risk of VTE who might benefit from primary thromboprophylaxis. In primary brain cancer patients, the risk of VTE is particularly high but likewise antithrombotic therapy is especially challenging in these patients.

Given the unmet medical need for biomarkers that anticipate patients at an especially high VTE risk, in this study we ventured into novel methods for profiling thrombosis‐associated phenotypes. Since miRNAs are reliably detectable in high‐grade glioma patient blood and since they can regulate mRNA expression in multiple cell types as well as systems of cells, they were a reasonable choice.

In addition to that, the specific biology of VTE development in high‐grade glioma patients further pointed towards a potential relevance of miRNAs. Previous research suggests that the pathophysiology of VTE in brain cancer involves platelet activation through a molecule called podoplanin, which is frequently expressed by brain tumour cells. Interestingly, we could show in a previous study that low platelet counts are linked to podoplanin expression and to high VTE risk in brain cancer patients,^5^ suggesting that high platelet activation due to podoplanin leads to platelet consumption. It would therefore be of interest to measure platelet activation in patients' blood samples to investigate potential associations with VTE risk. Unfortunately, platelet activation tests are difficult to conduct in patients, as standard platelet activation tests are insufficiently standardized and need fresh blood. Recently, however, miRNAs emerged as potential novel markers reflecting platelet activation that can be measured by standardized methods in stored plasma samples.^13^ As platelet activation might hence be involved in the pathophysiology of VTE in glioblastoma, this represented another argument to hypothesize that miRNAs might be differentially regulated in patients who did and who did not develop VTE later in their disease course.

Indeed, as main finding of the exploratory analysis in the current study, we found apparently differentially regulated miRNAs in patients with VTE compared to patients without VTE (although not statistically significant after adjustment for multiple testing). In detail, miR‐221‐3p seemed upregulated and the miRNAs hsa‐miR‐183‐5p, hsa‐miR‐454‐3p, hsa‐miR‐941, hsa‐let‐7d‐5p showed a probable downregulation in patients who developed VTE compared to patients who remained VTE free during follow‐up. Interestingly, pathway analyses of these differentially regulated miRNAs suggested a function of these miRNAs in glioma‐specific pathways, like p53 signalling or PI3K‐Akt signalling, rather than platelet‐specific or coagulation‐associated pathways. This indicates that their origin is rather the high‐grade glioma itself and not the platelets. In light of the fact that tumour‐derived free miRNAs are part of high‐grade glioma plasma, it could well be that they stem from the brain parenchyma‐contained tumour and then have an impact on coagulation processes in the periphery. This view is further supported by the fact that many of the differentially expressed miRNAs have already been implicated with the biology of various cancers.^22^, ^23^

A small number of previous studies have specifically investigated the role of miRNAs in cancer‐associated VTE. One study by Oto et al found that plasma miRNAs predict post‐surgical PE in patients with various types of glioma.^24^ In detail, in this study 6 miRNAs (miR‐363‐3p, miR‐93‐3p, miR‐22‐5p, miR‐451a, miR‐222‐3p and miR‐140‐3p), measured in blood samples taken before glioma surgery, were shown to predict post‐surgical PE. Interestingly, miR‐363‐3p was also found to be differentially regulated in patients with or without VTE in our study. Experimental studies showed that miR‐363‐3p supports glioma growth by inducing epithelial‐to‐mesenchymal transition, increasing migration and proliferation and reducing apoptosis.^25^ Other miRNAs identified as predictors of VTE in the study by Oto et al could not be confirmed in our study cohort. Differences between the study results might be caused by dissimilarities in the patient cohort, whereby the study by Oto et al included also lower‐grade gliomas, while our study concentrated on WHO grade IV glioma only. Furthermore, in the study by Oto et al. blood was taken before surgery, and patients were actively screened for PE 2–7 days after surgery. In contrast, in our study blood samples were taken after histological diagnosis of the tumour, i.e. after surgery/biopsy; and patients were followed for up to 2 years for symptomatic PE/DVT; no screening for PE or DVT was performed. Mechanisms of post‐surgical PE might be distinct from mechanisms of thromboembolism after the post‐surgical period and therefore, risk factors and biomarkers might differ. In another study, which was published as a congress abstract only, miRNAs were investigated as potential predictors of VTE in patients with colorectal cancer.^26^ In this study, a total of nine miRNAs were found to be differentially regulated in cases (cancer patients who developed VTE during a 6‐month observation period) versus controls (cancer patients who did not develop VTE during the follow‐up period): has‐miR‐4451, 942‐3p, 8063, 3132, 3118, 105‐5p 891‐5p, 200a‐5p and 6832‐3p. The miRNAs found in this study could not be confirmed in our study cohort; however, patient populations were different (colorectal cancer vs. glioblastoma) and mechanisms of VTE might be distinct between cancer types.

While our mapping of potentially VTE‐associated miRNAs of high‐grade glioma patients was the main study goal, three further worthwhile contributions were generated during the course of the research. First, we here provide one of the largest and deepest miRNome datasets of peripheral blood of high‐grade glioma patients—even without considering any reference to VTE and especially when considering that RNA sequencing was performed.^10^ The dataset has been uploaded and it is freely available for further research. When examining the dataset, miR‐451a was generally the most abundant miRNA. This is in line with previous glioblastoma research and seems to validate it.^27^ MiR‐451a has further been implicated in immunological processes^28^ and also erythrocyte and platelet biology.^29^ Additional research will have to clarify its role. Second, we describe that 51% of all miRNAs found were significantly correlated with the platelet count. Again, this substantiates similar prior observations^29^ and it represents an important caveat for all research that studies blood‐based miRNA signatures. And third, we provide a first indication of miRNAs associated with podoplanin, therewith contributing to the body of knowledge around the fundamental molecular biology processes underlying VTE formation.

Strengths of the current study include the embedding in a prospective observational study, i.e. the Vienna cancer and thrombosis study (CATS), which is a large study that was designed with the primary aim of identifying risk factors for VTE in patients with different types of cancer. Furthermore, miRNA sequencing was performed by a novel, robust and validated NGS microRNA discovery assay, using a standardized method that was recently published.^17^

A limitation of the current study is the restricted sample size, thus not allowing us to draw definitive conclusions about the respective miRNAs for the prediction of VTE in glioblastoma patients. However, glioblastoma is a very rare cancer type, and the full cohort used for the current nested matched case–control study consisted of a total number of 213 patients who were prospectively followed and of whom biological samples were available, which is arguably a substantial total cohort size. And, in terms of peripheral blood miRNA sequencing in high‐grade glioma patients, this cohort seems to be one of the largest so far. The nested matched case–control design was used to optimize efforts and to minimize costs. It generally worked very well as age‐ and sex‐matched controls without VTE could be identified for our VTE cases. Of note, the IDH1 mutation status was not used as a predefined matching criterium for our study, and the higher number of IDH positive brain tumours in the control group (3 patients, vs. 0 in the VTE group) might have had an influence. Thus, this slight imbalance could have potentially skewed the miRNA sequencing results. But since the three patients still only represent a minority and the vast majority (i.e. 87%) of the control group patients were IDH wildtype and therewith the same status as all the VTE patients, we deem this imbalance negligible. Cleary, further additional factors from the patient history like co‐morbidities could have had an impact on VTE risk, but it is nearly impossible to include the full complexity of a patient history in statistical algorithms like propensity‐score matching and we thus had to prioritize.

Another limitation of our study might be that blood was drawn into standard citrated blood tubes and plasma was generated by standard centrifugation steps without the use of any specific agents inhibiting platelet function, which was shown to cause artificial platelet activation during sample handling ex vivo and thereby influences the miRNA composition in plasma.^29^ However, we believe that a clinically relevant biomarker that might be used for the prediction of VTE in patients would require stability in conventional blood drawing methods.

In conclusion, several miRNAs showed a probable differential expression in patients who later developed VTE compared to patients who remained VTE free, and these miRNAs were mainly related to cancer‐associated pathways. Although a final confirmation is still needed as this was an exploratory screening (without multiple testing correction) we believe that our data provide meaningful new insights into miRNA regulation in high‐grade glioma patients. Further research is required to substantiate a role of miRNAs in the high thrombotic risk of glioblastoma patients. The comprehensive miRNome dataset we provide here will serve as a convenient starting point for that. Additionally, with the work shown here, we established the feasibility and usability of peripheral blood miRNomics for VTE biomarker research in neurooncological patients.

## AUTHOR CONTRIBUTIONS

**Friedrich Erhart:** Conceptualization (lead); funding acquisition (lead); project administration (equal); writing – original draft (lead); writing – review and editing (lead). **Georg Widhalm:** Conceptualization (supporting); writing – review and editing (supporting). **Barbara Kiesel:** Conceptualization (supporting); writing – review and editing (supporting). **Matthias Hackl:** Conceptualization (supporting); formal analysis (supporting); supervision (supporting); writing – review and editing (supporting). **Andreas Diendorfer:** Conceptualization (supporting); data curation (supporting); formal analysis (equal); writing – review and editing (supporting). **Matthias Preusser:** Conceptualization (supporting); writing – review and editing (supporting). **Karl Rössler:** Conceptualization (supporting); writing – review and editing (supporting). **Johannes Thaler:** Conceptualization (supporting); writing – review and editing (supporting). **Ingrid Pabinger:** Conceptualization (supporting); writing – review and editing (supporting). **Cihan Ay:** Conceptualization (supporting); writing – review and editing (supporting). **Julia Riedl:** Conceptualization (lead); data curation (equal); formal analysis (equal); methodology (lead); writing – original draft (lead); writing – review and editing (lead).

## FUNDING INFORMATION

This study was supported by the Austrian Research Promotion Agency (‘FFG Innovation voucher’, project number 43236429, granted to FE and GW). The Vienna Cancer and Thrombosis Study (CATS) was supported by funds of the ‘Oesterreichische Nationalbank’ (Anniversary Fund, project numbers 12739 and 14744).

## CONFLICT OF INTEREST STATEMENT

M.H. is employed by TAmiRNA GmbH and is a company shareholder. A.D. is employed by TAmiRNA GmbH. M.P. has received honoraria for lectures, consultation or advisory board participation from the following for‐profit companies: Bayer, Bristol‐Myers Squibb, Novartis, Gerson Lehrman Group (GLG), CMC Contrast, GlaxoSmithKline, Mundipharma, Roche, BMJ Journals, MedMedia, Astra Zeneca, AbbVie, Lilly, Medahead, Daiichi Sankyo, Sanofi, Merck Sharp & Dome, Tocagen, Adastra, Gan & Lee Pharmaceuticals. All other authors declare that the research was conducted in the absence of any other commercial, financial or personal relationships that could be construed as a potential competing interest.

## Supporting information


Appendix S1.

## Data Availability

The next‐generation miRNA sequencing data have been deposited to the NCBI Gene Expression Omnibus data repository with the accession number GSE222666—they are publicly available.
