# Supplementary material for: The plasma miRNome and venous thromboembolism in high‐grade glioma: miRNA Sequencing of a nested case–control cohort
Source: J Cell Mol Med. 2024 Apr 13;28(8):e18149. doi: 10.1111/jcmm.18149 (PMC11015389; doi:10.1111/jcmm.18149)
Supplement: Supplementary file 1 — Appendix S1. [file JCMM-28-e18149-s001.pdf]

Supplementary Figure 1: Analysis of LDH in all samples where the value had been measured as part of the routine patient blood monitoring (26/44, 59.1%). When comparing VTE cases with patients who did not develop VTE, no significant difference was registered, thus indicating that no significantly confounding hemolysis took place.

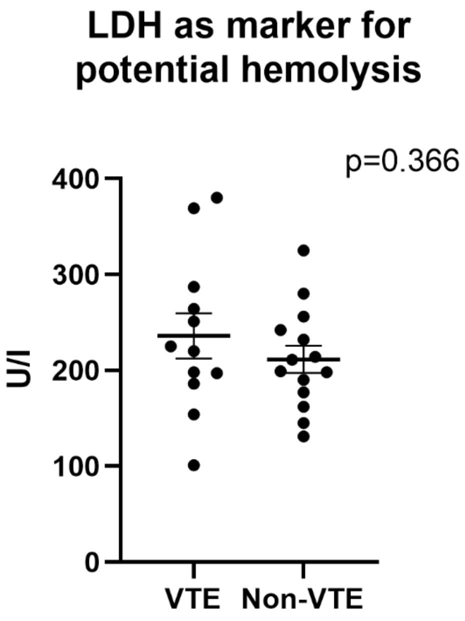

Supplementary Figure 2: Principal component analysis (PCA) from all samples and all miRNAs. No unanimous clustering of samples is seen.

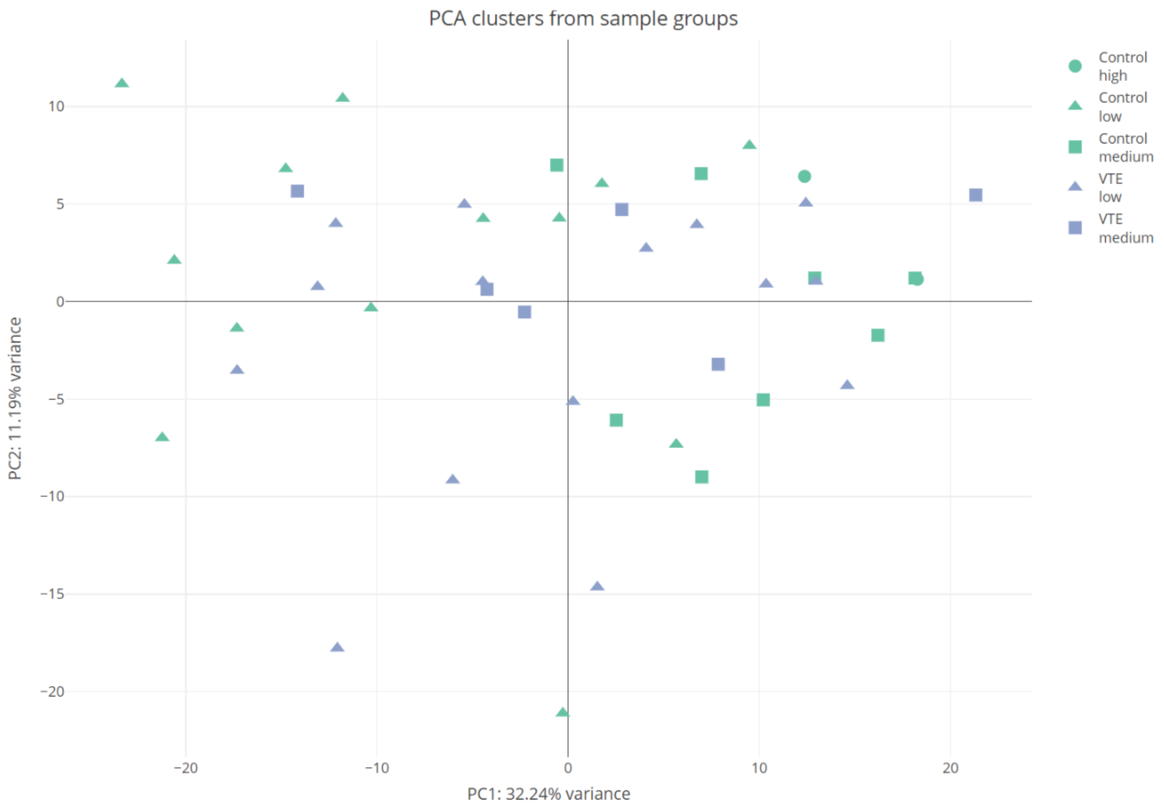

Supplementary Figure 3: T-stochastic neighbour embedding (t-SNE) from all samples and all miRNAs. t-SNE is a nonlinear dimensionality reduction technique well-suited for embedding high-dimensional data for visualization in a low-dimensional space (like 2 dimensions here). It models each high-dimensional object by a two- or three-dimensional point in such a way that similar objects are modelled by nearby points and dissimilar objects are modelled by distant points with high probability. No unanimous clustering of samples is seen.

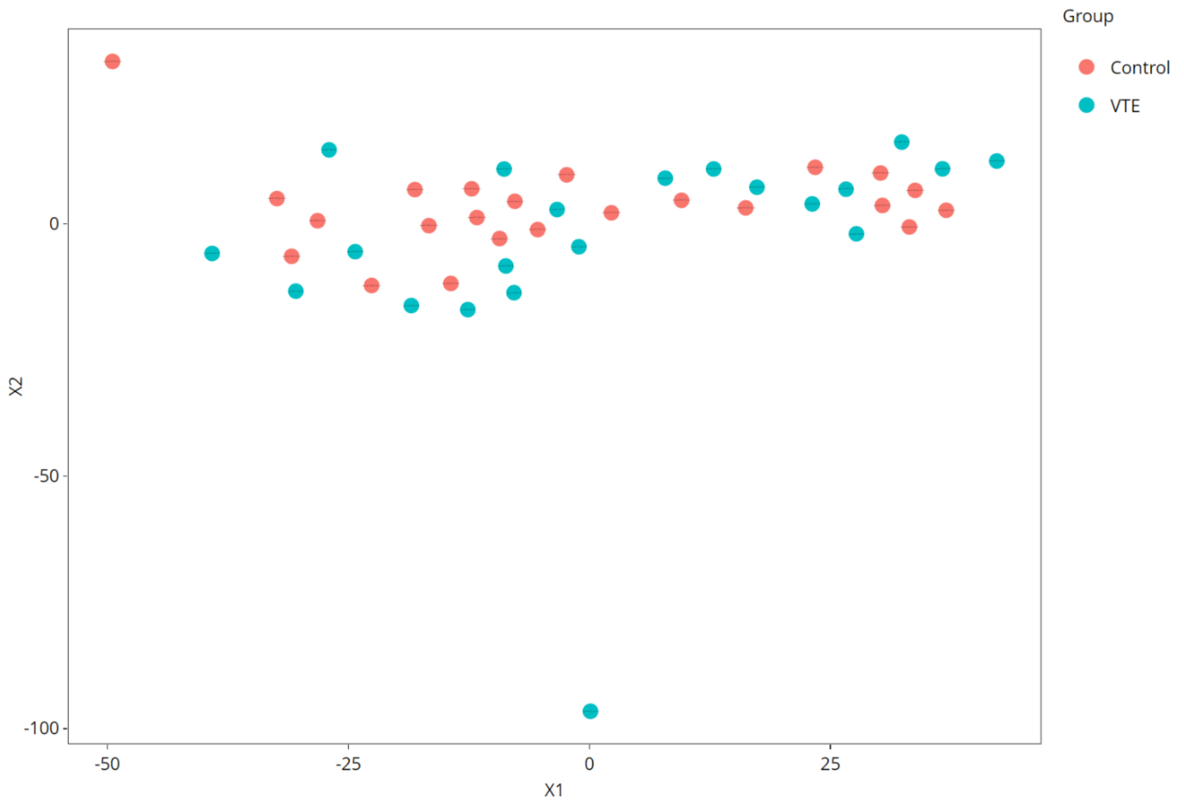

Supplementary Table 1. Pathway prediction analysis of up- und downregulated miRNAs in patients with podoplanin positive vs. podoplanin negative tumors (Tarbase v7.0*)*

**KEGG pathway**	**p-value**	**#genes**	**#miRNAs**
*Targets of miRNAs **down**regulated in patients with podoplanin positive vs. negative tumors*			
Fatty acid biosynthesis	3.11676347013e-14	4	2
Fatty acid metabolism	4.4528624749e-10	13	3
Hippo signaling pathway	8.78684615191e-07	40	5
Viral carcinogenesis	8.80463908464e-07	53	5
Protein processing in endoplasmic reticulum	1.7910970818e-05	48	4
*Targets of miRNAs **up**regulated in patients with podoplanin positive vs. negative tumors*			
Adherens junction	1.30095130887e-08	26	3
Bacterial invasion of epithelial cells	4.81625485254e-07	27	3
Proteoglycans in cancer	1.56919577627e-06	47	3
Circadian rhythm	8.86327565371e-06	15	3
Shigellosis	8.86327565371e-06	24	3
